# The complete mitochondrial genome of *Corythoxestis sunosei* (Lepidoptera: Gracillariidae) with phylogenetic consideration

**DOI:** 10.1080/23802359.2020.1790326

**Published:** 2020-07-15

**Authors:** Zhu-Ting Zhang, Jing Li, Dao-Chao Jin

**Affiliations:** a Provincial Key Laboratory for Agricultural Pest Management of Mountainous Regions, Institute of Entomology, Guizhou University, Guiyang, PR China; b School of Life and Health Science, Kaili University, Kaili, PR China

**Keywords:** *Corythoxestis sunosei*, Gracillariidae, mitogenome

## Abstract

The complete mitogenome of *Corythoxestis sunosei* (GenBank accession number MT611524) is 15,511 bp in length, and harbors 13 protein-coding genes (PCGs), 22 transfer RNA genes (tRNAs), two ribosomal RNA genes, and an A + T-rich region. The overall base composition is A (40.04%), C (10.64%), G (7.62%), and T (41.70%), showing AT-rich feature (81.74%). ATG, ATT, CGA were initiation codons and TAA and T were termination codons. All the 22 tRNAs displayed a common cloverleaf secondary structure, except for *trnS_1_* which lacked the dihydrouracil (DHU) arm. Phylogenetic tree based on 13 PCGs showed that *C. sunosei* has a close phylogenetic relationship with *Gibbovalva kobusi* and *Cameraria ohridella* and belongs to Gracillariidae.

The leaf-mining moth *Corythoxestis sunosei* belongs to the family Gracillariidae, which includes about 2556 described species in 151 genera (De Prins and De Prins 2019). *C. sunosei* was found for the first time mining the leaves of Chinese medical plant *Uncaria rhynchophylla* in China (Jiang et al. 2020). In this study, adult specimens of *C. sunosei* were collected from Jianhe County, Guizhou Province, China (N27°52′, E108°47′), and deposited in the insect specimen room of Shandong Normal University with an accession number SDNU. Ent005642–5653.

The complete mitogenome of *C. sunosei* (GenBank accession number MT611524) is 15,511 bp in length, and contains 13 protein-coding genes (PCGs), 22 transfer RNA genes (tRNAs), 2 ribosomal RNA genes (*16S rRNA* and *12S rRNA*), and an A + T-rich region (putative control region) (Boore 1999). The gene order and organization of *C. sunosei* were identical to those observed in other moth mitogenomes (Peng et al. 2017; Chen et al. 2019). The overall base composition of *C. sunosei* mitogenome were A (40.04%), C (10.64%), G (7.62%), and T (41.70%), with A + T content of 81.74%. The AT-skew and GC-skew of this mitogenome were −0.020 and −0.166, respectively. Fourteen genes (*trnQ, trnC, trnF, trnH, trnY, trnL_1_, trnP, trnV, nad1, nad4, nad4L, nad5,12S rRNA,* and *16S rRNA*) were encoded on the minority strand (N-strand) of the mitogenome, whereas the remaining genes were encoded on the majority strand (J-strand). Gene overlaps were present at 11 gene junctions and involved a total of 33 bp; the longest overlap (8 bp) is located between *trnW* and *trnC*. There are 10 intergenic spacer regions comprising a total of 121 bp with length varying from 1 to 56 bp. The largest intergenic spacer is located between *nad2* and *trnQ*. The length of 22 tRNAs varied from 61 bp (*trnC*) to 71 bp (*trnK*), and A + T content varied from 71.21% (*trnL_2_*) to 92.42% (*trnE*). All the 22 tRNAs displayed a common cloverleaf secondary structure, except for *trnS_1_* (AGN) which lacked the dihydrouracil (DHU) arm. The *16S rRNA* is located between *trnL_1_* and *trnV*, with length of 323 bp and A + T content of 86.47%. The *12S rRNA* is located between *trnV* and control region, with length of 720 bp and A + T content of 85.14%. The control region is located between *12S rRNA* and *trnM* is 735 bp in length with an A + T content of 94.97%.

The initial codons for 12 PCGs were cononical putative start condons ATN (ATG for *cox2*, *cox3*, *cob, atp6*, *nad1*, *nad4*, *nad4L*, and *nad6*; ATT for *atp8*, *nad2*, *nad3*, and *nad5*), whereas *cox1* use CGA as start codon. Ten PCGs terminate with the stop codon TAA; however, *cob*, *nad4*, and *nad5* use incomplete T as stop codon, respectively. Based on the concatenated amino acid sequences of 13 PCGs, the neighbor-joining method was used to construct phylogenetic relationship of *C. sunosei* and 15 other moths with MEGA7 (Kumar et al. 2016). The result showed that *C. sunosei* has a close phylogenetic relationship with *Gibbovalva kobusi* and *Cameraria ohridella* (Figure 1), which agree with the morphology taxonomy of the family Gracillariidae.

**Figure 1.**
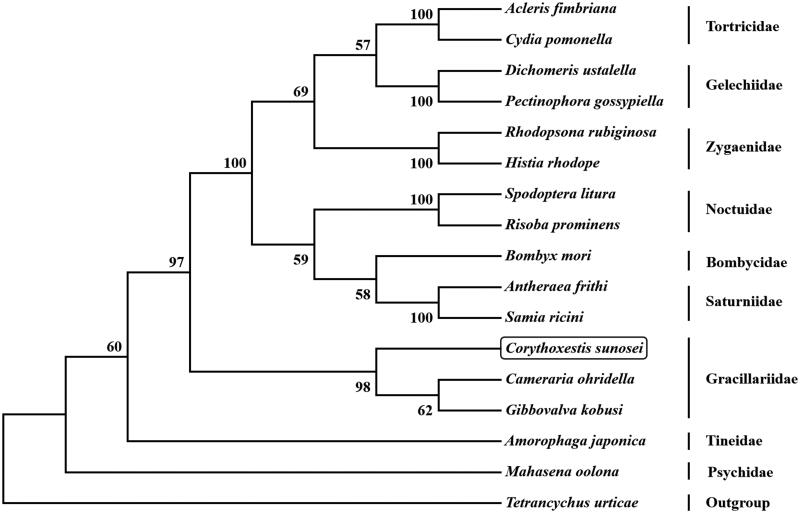
Phylogenetic tree showing the relationship between *Corythoxestis sunosei* and 15 other mothes based on neighbor-joining method. GenBank accession numbers used in this study are the following: *Acleris fimbriana* (HQ662522)*, Amorophaga japonica* (MH823253), *Antheraea frithi* (NC_027071), *Bombyx mori* (NC_002355), *Cameraria ohridella* (KJ508042), *Corythoxestis sunosei* (MT611524), *Cydia pomonella* (JX407107), *Dichomeris ustalella* (KU366706), *Gibbovalva kobusi* (MK956103), *Histia rhodope* (MF542357), *Mahasena oolona* (NC_036410), *Pectinophora gossypiella* (KM225795), *Rhodopsona rubiginosa* (KM244668), *Risoba prominens* (KJ396197), *Samia ricini* (NC_017869), *Spodoptera litura* (KF701043), *Tetrancychus urticae* (EU345430). *T. urticae* was used as an outgroup. The moth determined in this study is boxed.

## Data Availability

The data that support the findings of this study are openly available in the GenBank at https://www.ncbi.nlm.nih.gov/genbank/, reference number MT611524.
